# Supplementary figures and images for: Deciphering Active Prophages from Metagenomes
Source: mSystems. 2022 Mar 24;7(2):e00084-22. doi: 10.1128/msystems.00084-22 (PMC9040807; doi:10.1128/msystems.00084-22)

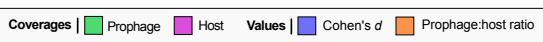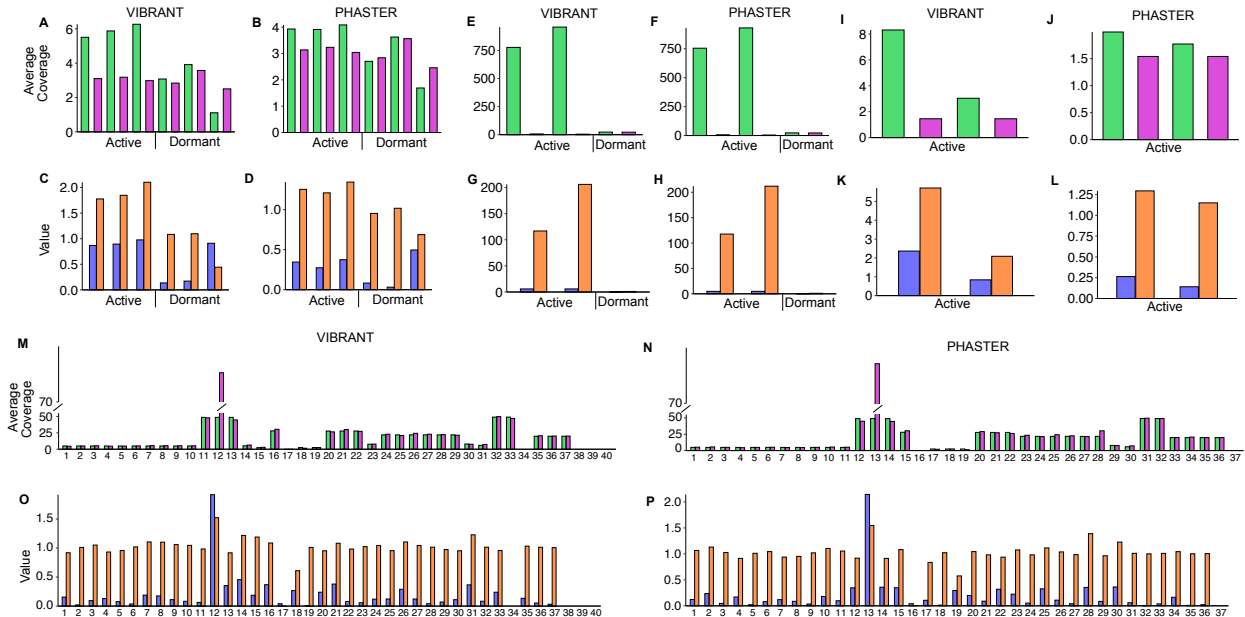

Supplement: FIG S1 [file msystems.00084-22-sf001.pdf]

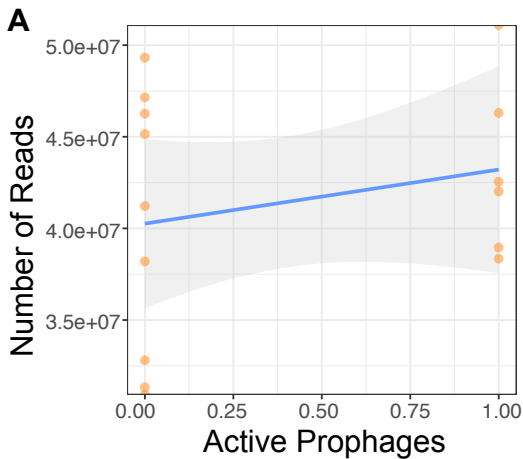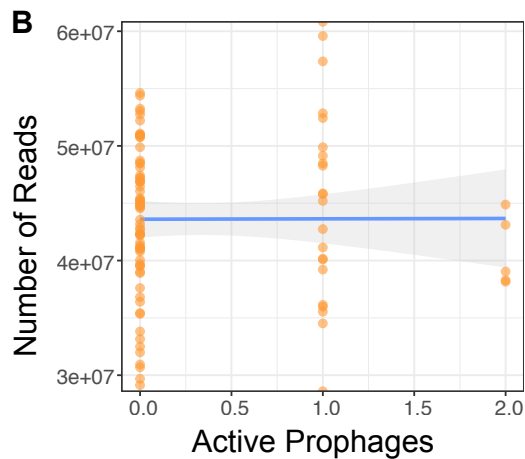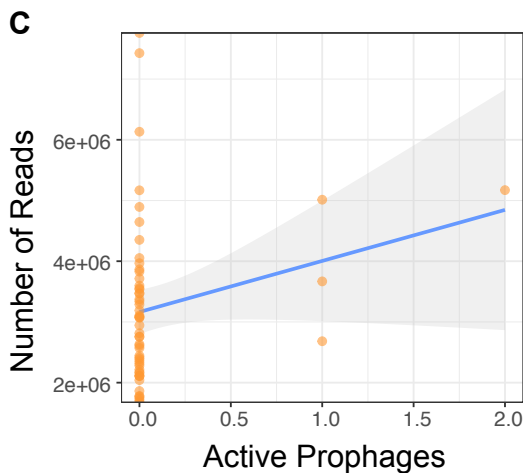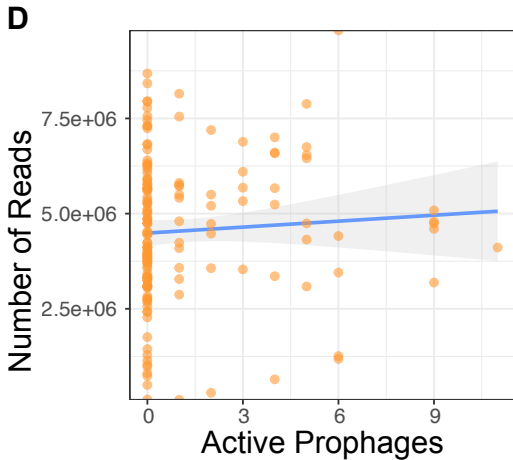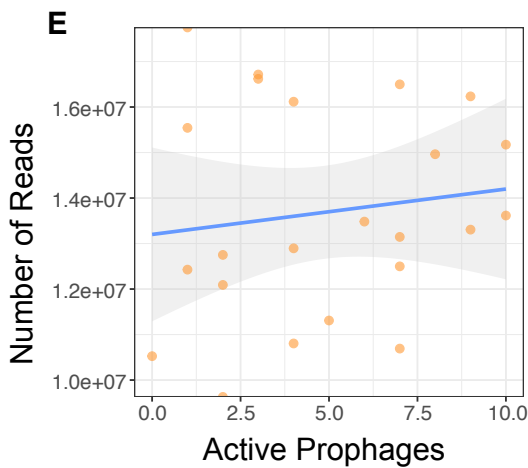

Supplement: FIG S4 [file msystems.00084-22-sf004.pdf]

## Active prophages across samples

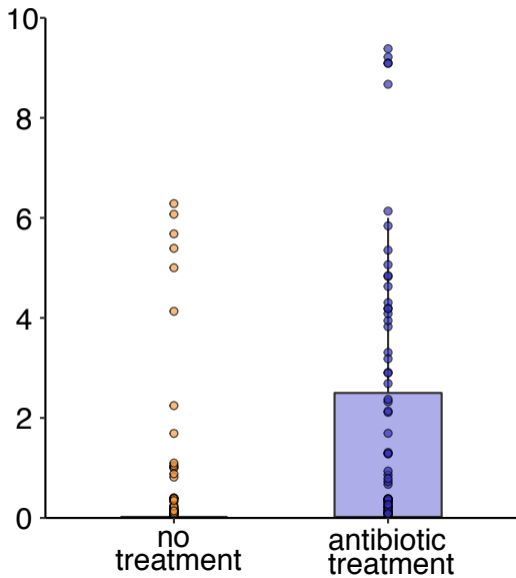

# B

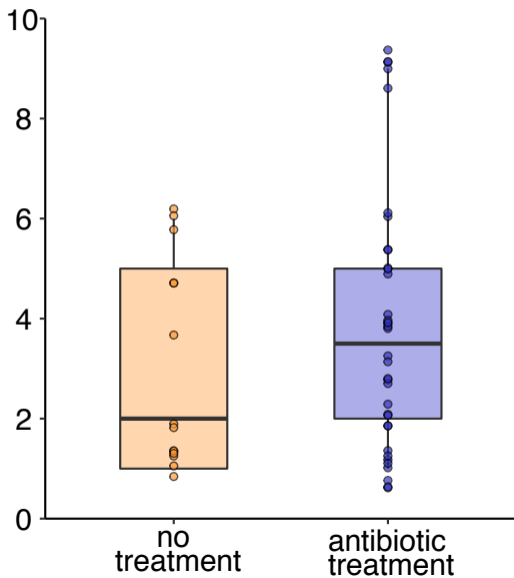

Supplement: FIG S2 [file msystems.00084-22-sf002.pdf]
