# Supplementary material for: Deciphering Active Prophages from Metagenomes
Source: mSystems. 2022 Mar 24;7(2):e00084-22. doi: 10.1128/msystems.00084-22 (PMC9040807; doi:10.1128/msystems.00084-22)
Supplement: FIG S3 [file msystems.00084-22-sf003.pdf]

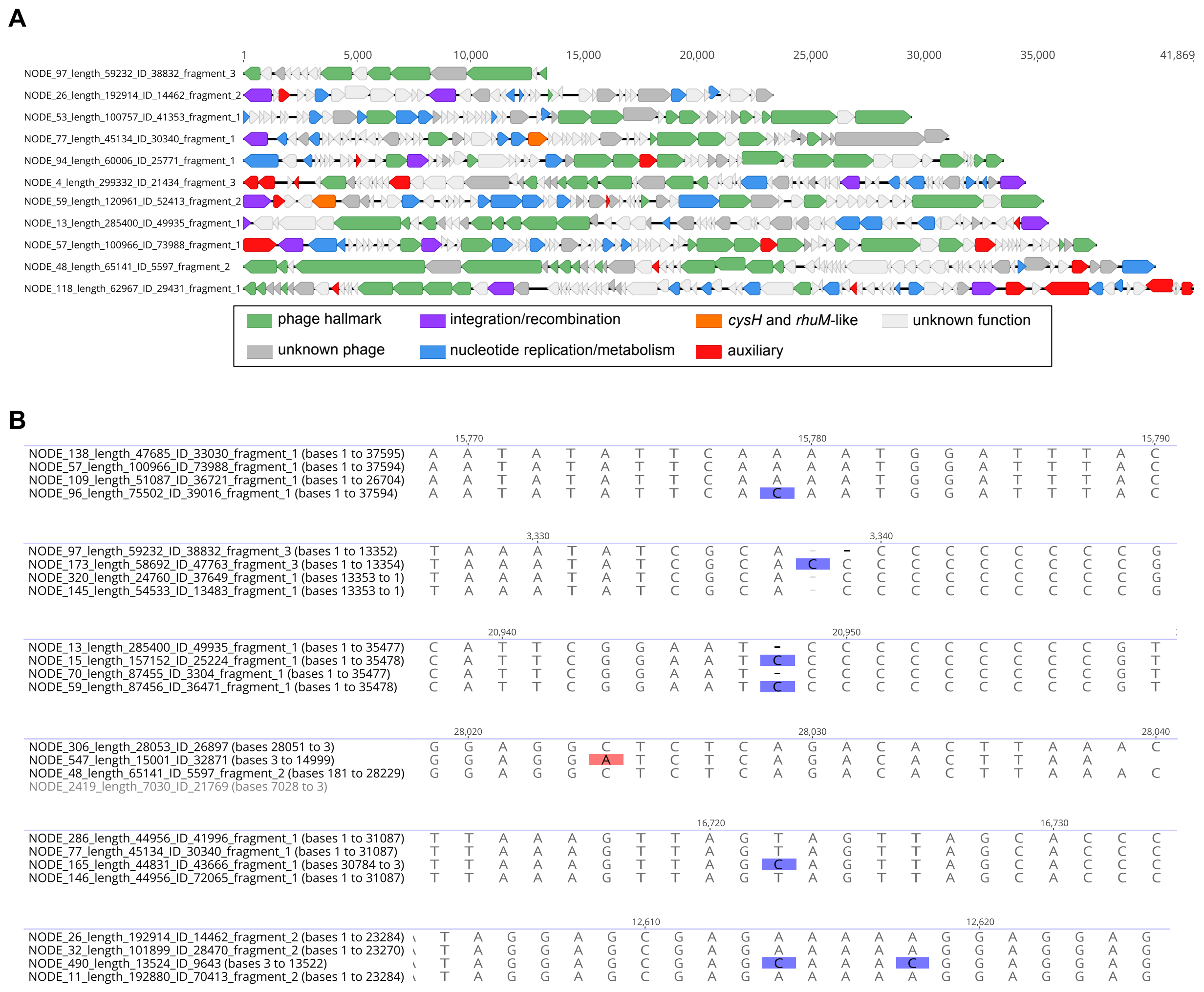
A
1
5,000
10,000
15,000
20,000
25,000
30,000
35,000
41,869
NODE_97_length_59232_ID_38832_fragment_3
NODE_26_length_192914_ID_14462_fragment_2
NODE_53_length_100757_ID_41353_fragment_1
NODE_77_length_45134_ID_30340_fragment_1
NODE_94_length_60006_ID_25771_fragment_1
NODE_4_length_299332_ID_21434_fragment_3
NODE_59_length_120961_ID_52413_fragment_2
NODE_13_length_285400_ID_49935_fragment_1
NODE_57_length_100966_ID_73988_fragment_1
NODE_48_length_65141_ID_5597_fragment_2
NODE_118_length_62967_ID_29431_fragment_1
phage hallmark
integration/recombination
cysH and rhuM-like
unknown function
unknown phage
nucleotide replication/metabolism
auxiliary
B
15,770
15,780
15,790
NODE_138_length_47685_ID_33030_fragment_1 (bases 1 to 37595) A A T A T A T T C A A A A T G G A T T T A C
NODE_57_length_100966_ID_73988_fragment_1 (bases 1 to 37594) A A T A T A T T C A A A A T G G A T T T A C
NODE_109_length_51087_ID_36721_fragment_1 (bases 1 to 26704) A A T A T A T T C A A A A T G G A T T T A C
NODE_96_length_75502_ID_39016_fragment_1 (bases 1 to 37594) A A T A T A T T C A C A A T G G A T T T A C
3,330
3,340
NODE_97_length_59232_ID_38832_fragment_3 (bases 1 to 13352) T A A A T A T C G C A – – C C C C C C C C G
NODE_173_length_58692_ID_47763_fragment_3 (bases 1 to 13354) T A A A T A T C G C A C C C C C C C C C C G
NODE_320_length_24760_ID_37649_fragment_1 (bases 13353 to 1) T A A A T A T C G C A – C C C C C C C C C G
NODE_145_length_54533_ID_13483_fragment_1 (bases 13353 to 1) T A A A T A T C G C A – C C C C C C C C C G
20,940
20,950
NODE_13_length_285400_ID_49935_fragment_1 (bases 1 to 35477) C A T T C G G A A T – C C C C C C C C C G T
NODE_15_length_157152_ID_25224_fragment_1 (bases 1 to 35478) C A T T C G G A A T C C C C C C C C C C G T
NODE_70_length_87455_ID_3304_fragment_1 (bases 1 to 35477) C A T T C G G A A T – C C C C C C C C C G T
NODE_59_length_87456_ID_36471_fragment_1 (bases 1 to 35478) C A T T C G G A A T C C C C C C C C C C G T
28,020
28,030
28,040
NODE_306_length_28053_ID_26897 (bases 28051 to 3) G G A G G C T C T C A G A C A C T T A A A C
NODE_547_length_15001_ID_32871 (bases 3 to 14999) G G A G G A T C T C A G A C A C T T A A
NODE_48_length_65141_ID_5597_fragment_2 (bases 181 to 28229) G G A G G C T C T C A G A C A C T T A A A C
NODE_2419_length_7030_ID_21769 (bases 7028 to 3)
16,720
16,730
NODE_286_length_44956_ID_41996_fragment_1 (bases 1 to 31087) T T A A A G T T A G T A G T T A G C A C C C
NODE_77_length_45134_ID_30340_fragment_1 (bases 1 to 31087) T T A A A G T T A G T A G T T A G C A C C C
NODE_165_length_44831_ID_43666_fragment_1 (bases 30784 to 3) T T A A A G T T A G C A G T T A G C A C C C
NODE_146_length_44956_ID_72065_fragment_1 (bases 1 to 31087) T T A A A G T T A G T A G T T A G C A C C C
12,610
12,620
NODE_26_length_192914_ID_14462_fragment_2 (bases 1 to 23284) T A G G A G C G A G A A A A A G G A G G A G
NODE_32_length_101899_ID_28470_fragment_2 (bases 1 to 23270) T A G G A G C G A G A A A A A G G A G G A G
NODE_490_length_13524_ID_9643 (bases 3 to 13522) T A G G A G C G A G C A A A C G G A G G A G
NODE_11_length_192880_ID_70413_fragment_2 (bases 1 to 23284) T A G G A G C G A G A A A A A G G A G G A G
